# Supplementary material for: Snakebite envenomations and access to treatment in communities of two indigenous areas of the Western Brazilian Amazon: A cross-sectional study
Source: PLoS Negl Trop Dis. 2023 Jul 13;17(7):e0011485. doi: 10.1371/journal.pntd.0011485 (PMC10368234; doi:10.1371/journal.pntd.0011485)
Supplement: Attachment — Submitted filename: COMMENTS.docx [file pntd.0011485.s003.docx]

**Comments**

General comments: This is an important topic and is relevant in generating data on snakebite envenoming, given that hospital data alone does not tell the whole story of SBE globally. However, below are a few specific comments that should help strengthen the manuscript's quality.

**Specific comments:**

**Introduction**: The recount of the existing literature on the global burden of SBE, policies to mitigate it, local Brazilian Amazonian literature on the burden, paucity of data on SBE, antivenom crises, and barriers to access to medical facility care regarding snakebite is adequate. However, I struggled to understand the specific objectives of the study from the stated study aim.

Aim – page 7, lines 132-134: The specific objectives of this study may need to be modified to improve their specificity, clarity, measurability and reproducibility. This way, the reader knows the information to expect in the results and discussion sections of the paper. Thus, I offer the following suggestions.

(1) The statement "to estimate SBE underreporting" may need to be modified: Given that some of the participants in this study accessed orthodox care, which should have been documented and, as such, are reported cases, it will be inappropriate to describe them as under-reporting. I suggest rephrasing this objective, e.g. to read: "to estimate SBE reported by the community-dwellers….."
(2) For clarity, I suggest that you include that you are "assessing participants' health care seeking behaviour following a snakebite".
(3) Analyze barriers that prevent victims from obtaining healthcare in indigenous communities located in two indigenous health districts, in the Western Brazilian Amazon.

**Methods**: How did the authors arrive at a sample size of 187?

**Results:**

1. Table 1, schooling the second and third columns don't total 100%. Similarly, cross-check occupation, religion, etc. Please cross-check the data in all tables.
2. Table 4: the legend to this table is confusing and should be rephrased to reflect that it contains factors associated with lack of access to healthcare.

**Discussion**:

(1) What were the limitations of this study?
(2) There were no clear statements about the policy implications of the study's outcome.
