# Supplementary material for: Snakebite envenomations and access to treatment in communities of two indigenous areas of the Western Brazilian Amazon: A cross-sectional study
Source: PLoS Negl Trop Dis. 2023 Jul 13;17(7):e0011485. doi: 10.1371/journal.pntd.0011485 (PMC10368234; doi:10.1371/journal.pntd.0011485)
Supplement: Attachment — Submitted filename: Rebuttal letter.docx [file pntd.0011485.s004.docx]

Dear Editor,
 
Thank you very much for the chance to resubmit the manuscript "Snakebite Envenomations and Access to Treatment in Communities of Two Indigenous Areas of the Western Brazilian Amazon: a Cross-Sectional Study" for consideration at PLOS Neglected Tropical Diseases. In this new version, we have tried to improve the manuscript by considering all reviewers' comments.

Reviewer #1: - the study objectives could be made clearer
- the study design is appropriate for the stated objectives
- no clear explanation was given for the sample size
- there was statistical analysis to support conclusions
- The ethical concerns were well addressed

RESPONSE: Thanks for the comments.

In this cross-sectional study, a non-probability sampling method was used to contact participants identified from SBE cases reported to the Indigenous Health Care Information System (*Sistema de Informação da Atenção à Saúde Indígena*; SIASI), cases identified by the indigenous health agents and other health professionals working in the villages, and by using a chain-referral (snowball) sampling technique, in which previously included participants in each village identified other eligible subjects from among their acquaintances [19].

This explanation was given in this version of the manuscript (lines 212-218).

Reviewer #2: No comments under the methods as the manuscript has well described the objectives and appropriate study design, selection of samples, defining population and appropriate analysis. No ethical issues have been noted since the authors have obtained the ethical approval.
RESPONSE: Thanks for the comments.

Reviewer #3: •  This study has a clear objective and reports relevant data on SBE on two indigenous populations. However, it is not clear how the estimation of underreporting was conducted from a statistical point of view.  The characteristics of those who did not receive medical attention after the snakebite are clearly stated, but it is not clear if all of them are not-reported cases.  I recommend including or changing the aim of the study to a more descriptive perspective on the management of SBE in two indigenous populations.
RESPONSE: Thanks for the comments. The reviewer is entirely correct. In the new version of the manuscript, we do not propose estimating underreporting, but only talk about 'access' and 'non-access' to the health system.

We changed the aim of the study as recommended.

•       The authors need to clarify if data collection was conducted using a survey or an interview (See lines 212 and 233).
RESPONSE: Thanks for the comment. We don’t use the term ‘survey’ in the revised version of the manuscript.

•       Lines 256 to 282 provide a detailed description of some characteristics of these populations; however, I recommend including part of this information in the introduction section. Also, it can be used in the methods section to briefly describe variables such as type of housing, occupation, and the characteristics of the place where the snakebite occurred.
RESPONSE: However, we ask the reviewer to keep the description of the area as it is in the original manuscript, since this information was collected in the field work itself, and not from references to previous studies, to be presented in the Introduction.

--------------------
Reviewer #1: - the analysis matched the analysis plan
- the results were clearly and completely presented
RESPONSE: Thanks for the comment.

Reviewer #2: No specific comments results, analysis, presentation, figures and images.
RESPONSE: Thanks for the comment.

Reviewer #3: •  Tables 1 and 2 allow an adequate description of the population.  Moreover, Table 3 describes relevant information on the use of indigenous medicine as a treatment by study participants.
•       I recommend including relevant data from Table 4 in the text.

RESPONSE: In the multivariate analysis, living in Upper Rio Negro SIHD was independently associated to the lack of access to healthcare [aOR 19.33 (95%CI=4.11-90.85); P-value<0.01] (Table 4).

This sentence shows the only variable associated to access to care.

•       Pictures in Figure 3 can be reduced and present just those that illustrate the housing, transportation, and lifestyle characteristics of the study population.

RESPONSE: Thanks for the comment. We ensure that all the pictures used in Fig. 3 are important to illustrate the environment, housing, transportation, and lifestyle characteristics of the study population.

•       The use of the abbreviations SBE and SBEs is confusing throughout the whole document.  Please review.

RESPONSE: A standardization was made accordingly.

•       Line 506: I assume the abbreviation AV refers to antivenoms,  as it was not previously mentioned in the document.

RESPONSE: AV was changed to ‘antivenom’ in the revised version.

•       Line 339: is the age range 49-59 or 40-59 years old, as stated in Table 1?

RESPONSE: ‘49-59’ was changed to ‘40-59’.

--------------------
Reviewer #1: - conclusion supported the data presented
- there were no clearly stated limitations of the study
- the authors discussed the data appropriately
- the public health relevance of the study outcome was not clearly stated
RESPONSE: Thanks for the comments. Suggestions were added accordingly.

Reviewer #2: Yes agree. Conclusions were drawn from the analyzed results. Limitation of the study have been discussed adequately.
RESPONSE: Thanks for the comments.

Reviewer #3: •  Study limitations are not clearly stated in the document. A discussion on the generalizability and implications of results for the indigenous population is not presented.

In this study, we adopted a cross-sectional approach with a non-probabilistic sampling based on the ease of access and existing contact within the population of interest. However, some indigenous villages were not included due to difficulties in terms of access, which prevented the study from having a greater number of participants. These more distant villages are precisely the ones that are most likely to have a greater number of bad cases and less favorable outcomes. As a result, our sampling method impairs the generalizability of our results. Moreover, unfortunately, the number of inhabitants per community was not obtained, which did not allow the calculation of the prevalence of snakebites in this population. The study also has a limitation in that it does not represent the medical reality of all indigenous ethnicities in the Brazilian Amazon, due to their cultural differences and possibilities of accessing the health system. Finally, the collection of information in this study depends on the memory of the participants, who in many cases were already elderly or had suffered their SBE more than a decade ago.

We added this paragraph at the end of the Discussion section.

•       The conclusion does not refer to the relevance of study findings to promote and recommend actions to access antivenoms soon after a snakebite occurs and to bring traditional medicine and Western medical treatment together. As stated in lines 484-487.

RESPONSE: Conclusion was changed accordingly.

--------------------
Reviewer #1: - there will be a need for English language editing as the grammatical style of the authors made understanding their thought flow difficult.
RESPONSE: English language editing was made by a native speaker.
--------------------
Reviewer #1: Comments
General comments: This is an important topic and is relevant in generating data on snakebite envenoming, given that hospital data alone does not tell the whole story of SBE globally. However, below are a few specific comments that should help strengthen the manuscript's quality.
Specific comments:
Introduction: The recount of the existing literature on the global burden of SBE, policies to mitigate it, local Brazilian Amazonian literature on the burden, paucity of data on SBE, antivenom crises, and barriers to access to medical facility care regarding snakebite is adequate. However, I struggled to understand the specific objectives of the study from the stated study aim.
Aim – page 7, lines 132-134: The specific objectives of this study may need to be modified to improve their specificity, clarity, measurability and reproducibility. This way, the reader knows the information to expect in the results and discussion sections of the paper. Thus, I offer the following suggestions. 
(1)     The statement "to estimate SBE underreporting" may need to be modified: Given that some of the participants in this study accessed orthodox care, which should have been documented and, as such, are reported cases,  it will be inappropriate to describe them as under-reporting. I suggest rephrasing this objective, e.g. to read: "to estimate SBE reported by the community-dwellers….."
(2)     For clarity, I suggest that you include that you are "assessing participants' health care seeking behaviour following a snakebite".
(3)     Analyze barriers that prevent victims from obtaining healthcare in indigenous communities located in two indigenous health districts, in the Western Brazilian Amazon.

RESPONSE: Thanks for the comments. The reviewer is entirely correct. In the new version of the manuscript, we do not propose estimating underreporting, but only talk about 'access' and 'non-access' to the health system.

We changed the aim of the study as recommended, using the suggestion 3.

Methods: How did the authors arrive at a sample size of 187?

RESPONSE: Thanks for the comments.

In this survey, a non-probability sampling method was used to contact participants identified from SBE cases reported to the Indigenous Health Care Information System (*Sistema de Informação da Atenção à Saúde Indígena*; SIASI), cases identified by the indigenous health agents and other health professionals working in the villages, and by using a chain-referral (snowball) sampling technique, in which previously included participants in each village identified other eligible subjects from among their acquaintances [19].

This explanation was given in this version of the manuscript (lines 212-218).

Results:
1.      Table 1, schooling the second and third columns don't total 100%. Similarly, cross-check occupation, religion, etc. Please cross-check the data in all tables.

RESPONSE: As recommended, we cross-checked the data in all tables.

2.      Table 4: the legend to this table is confusing and should be rephrased to reflect that it contains factors associated with lack of access to healthcare.

RESPONSE: Legend of Table 4 was rephrased as recommended.

Discussion:
(1)     What were the limitations of this study?

In this study, we adopted a cross-sectional approach with a non-probabilistic sampling based on the ease of access and existing contact within the population of interest. However, some indigenous villages were not included due to difficulties in terms of access, which prevented the study from having a greater number of participants. These more distant villages are precisely the ones that are most likely to have a greater number of bad cases and less favorable outcomes. As a result, our sampling method impairs the generalizability of our results. Moreover, unfortunately, the number of inhabitants per community was not obtained, which did not allow the calculation of the prevalence of snakebites in this population. The study also has a limitation in that it does not represent the medical reality of all indigenous ethnicities in the Brazilian Amazon, due to their cultural differences and possibilities of accessing the health system. Finally, the collection of information in this study depends on the memory of the participants, who in many cases were already elderly or had suffered their SBE more than a decade ago.

We added this paragraph at the end of the Discussion section.

(2)     There were no clear statements about the policy implications of the study's outcome.

RESPONSE: Thanks for the comment. In this sense, we added some sentences at the end of the Conclusion section.

Reviewer #2: Include the total number of people included in this study 187 under the "Methods/principal findings" of the ABSTRACT.

RESPONSE:

Reviewer #3: This study details characteristics and information on how SBE is treated by two indigenous communities, as well as different factors that can facilitate or difficult access to treatment.  However, the objective of this study is just a part of the main focus of the discussion observed throughout the document.
RESPONSE: Thanks for the comments. The reviewer is entirely correct. In the new version of the manuscript, we do not propose estimating underreporting, but only talk about 'access' and 'non-access' to the health system.

We changed the aim of the study as recommended, using the suggestion given by the Reviewer 1.

In addition, I suggest analyzing study limitations and the possibility of generalized results to these indigenous communities.

In this study, we adopted a cross-sectional approach with a non-probabilistic sampling based on the ease of access and existing contact within the population of interest. However, some indigenous villages were not included due to difficulties in terms of access, which prevented the study from having a greater number of participants. These more distant villages are precisely the ones that are most likely to have a greater number of bad cases and less favorable outcomes. As a result, our sampling method impairs the generalizability of our results. Moreover, unfortunately, the number of inhabitants per community was not obtained, which did not allow the calculation of the prevalence of snakebites in this population. The study also has a limitation in that it does not represent the medical reality of all indigenous ethnicities in the Brazilian Amazon, due to their cultural differences and possibilities of accessing the health system. Finally, the collection of information in this study depends on the memory of the participants, who in many cases were already elderly or had suffered their SBE more than a decade ago.

We added this paragraph at the end of the Discussion section.

A final word of advice, since I had trouble following some sentences, I recommend a detailed review of the manuscript for grammar consistency.
RESPONSE: English language editing was made by a native speaker.
