# Supplementary material for: Snakebite envenomations and access to treatment in communities of two indigenous areas of the Western Brazilian Amazon: A cross-sectional study
Source: PLoS Negl Trop Dis. 2023 Jul 13;17(7):e0011485. doi: 10.1371/journal.pntd.0011485 (PMC10368234; doi:10.1371/journal.pntd.0011485)
Supplement: Attachment — Submitted filename: Rebuttal letter R2, survey.docx [file pntd.0011485.s005.docx]

Dear Editor,
 
Thank you very much for the chance to resubmit the manuscript "Snakebite Envenomations and Access to Treatment in Communities of Two Indigenous Areas of the Western Brazilian Amazon: a Cross-Sectional Study" for consideration at PLOS Neglected Tropical Diseases. In this new version, we have tried to improve the manuscript by considering all reviewers' comments.

Reviewer #1: How did the authors estimate the sample size?
RESPONSE: Thanks for the comments.

In this cross-sectional study, a non-probability sampling method was used to contact participants identified from SBE cases reported to the Indigenous Health Care Information System (*Sistema de Informação da Atenção à Saúde Indígena*; SIASI), cases identified by the indigenous health agents and other health professionals working in the villages, and by using a chain-referral (snowball) sampling technique, in which previously included participants in each village identified other eligible subjects from among their acquaintances [19].

This explanation was given in this version of the manuscript (lines 212-218).

Reviewer #2: -Are the objectives of the study clearly articulated with a clear testable hypothesis stated? Yes
-Is the study design appropriate to address the stated objectives? Yes
-Is the population clearly described and appropriate for the hypothesis being tested? Yes
-Is the sample size sufficient to ensure adequate power to address the hypothesis being tested? Yes
-Were correct statistical analysis used to support conclusions? Yes
-Are there concerns about ethical or regulatory requirements being met? Yes
RESPONSE: Thanks for the comments.

Reviewer #3: The population is clearly stated and described; for me, the objective of this study is to describe the lack of access to healthcare in these two populations, as stated in lines 287-292, and not an estimation of underreporting.
RESPONSE: Thanks for the comments. We changed the objective as recommended.

Reviewer #1: The issues highlighted in my comments about the Tables were not addressed.

Thanks for the comments.

On the comments of Revision 1:

RESPONSE 1: In the multivariate analysis, living in Upper Rio Negro SIHD was independently associated to the lack of access to healthcare [aOR 19.33 (95%CI=4.11-90.85); P-value<0.01] (Table 4).

This sentence shows the only variable associated to access to care and wwas included in the text.

RESPONSE 2: Line 339: is the age range 49-59 or 40-59 years old, as stated in Table 1?

‘49-59’ was changed to ‘40-59’.

RESPONSE 3: Table 1, schooling the second and third columns don't total 100%. Similarly, cross-check occupation, religion, etc. Please cross-check the data in all tables.

As recommended, we cross-checked the data in all tables.

RESPONSE 4: Table 4: the legend to this table is confusing and should be rephrased to reflect that it contains factors associated with lack of access to healthcare.

Legend of Table 4 was rephrased as recommended.

Reviewer #2: -Does the analysis presented match the analysis plan? Yes
-Are the results clearly and completely presented? Yes
-Are the figures (Tables, Images) of sufficient quality for clarity? Yes

RESPONSE: Thanks for the comments.

Reviewer #3: - Line 338: SBEs were reported or happened?
- Review lines 353 and 354
- Review the words "high" in line 430, and "even though" in line 434.
- Some ideas seem to be incomplete: lines 438-440, 454-456, 501-503
- Line 478: I assume the abbreviation AV refers to antivenoms, as it was not previously mentioned in the document.
- Throughout the document review the use of "peoples"; people is a plural form and does not require the "s". I suggest using population instead in most cases.
RESPONSE: Thanks for the comments. Changes were made accordingly.
--------------------

Conclusions

Reviewer #1: (No Response)

Reviewer #2: -Are the conclusions supported by the data presented? Yes
-Are the limitations of analysis clearly described? Yes
-Do the authors discuss how these data can be helpful to advance our understanding of the topic under study? Yes
-Is public health relevance addressed? Yes

RESPONSE: Thanks for the comments.

Reviewer #3: - Study limitations are still not clearly stated in the document.
- A discussion on the generalizability and implications of results for the indigenous population is not presented.

RESPONSE: In this study, we adopted a cross-sectional approach with a non-probabilistic sampling based on the ease of access and existing contact within the population of interest. However, some indigenous villages were not included due to difficulties in terms of access, which prevented the study from having a greater number of participants. These more distant villages are precisely the ones that are most likely to have a greater number of bad cases and less favorable outcomes. As a result, our sampling method impairs the generalizability of our results. Moreover, unfortunately, the number of inhabitants per community was not obtained, which did not allow the calculation of the prevalence of snakebites in this population. The study also has a limitation in that it does not represent the medical reality of all indigenous ethnicities in the Brazilian Amazon, due to their cultural differences and possibilities of accessing the health system. Finally, the collection of information in this study depends on the memory of the participants, who in many cases were already elderly or had suffered their SBE more than a decade ago.

We added this paragraph at the end of the Discussion section.

- Lines 543-547: This conclusion does not seem to come from the study design.

RESPONSE: Thanks for the comment. Sentence was deleted.

--------------------

Editorial and Data Presentation Modifications

Reviewer #1: (No Response)

Reviewer #2: I agree with all the modifications have been done to the revised version of the manuscript and I would recommend to accept this after the editorial decision.

RESPONSE: Thanks for the comments.

Reviewer #3: (No Response)

Summary and General Comments

Reviewer #1: 1) I am not sure in PLos journals, authors are not allowed to highlight corrections of queries from reviewers in their manuscript.
2) After painstakingly, going through the revised manuscript, i found that the authors did not respond to the comments regarding the issues I raised during the first submission.
3) Without any response from the authors I am constrained to recommend a revision
RESPONSE: The reviewer is totally correct.

We believe that there was some mistake and perhaps the earlier version of the manuscript (seen by the reviewer rather than the R1 version) was kept in the journal system and reviewer 1 did not see the version reviewed by the authors. In any case, we replied again and thank you for the comments that allowed us to improve the quality of the manuscript.

Reviewer #2: I agree with all the modifications have been done to the revised version of the manuscript and I would recommend to accept this after the editorial decision.

RESPONSE: Thanks for the comments.

Reviewer #3: I suggest reviewing the objective as I still consider the aim was to present data on access to healthcare more than identifying underreporting.  In addition, I suggest analyzing study limitations. Grammar consistency is better in this reviewed version, however, I still had trouble following some sentences.

RESPONSE 1: Thanks for the comments.

RESPONSE 2: A new English language editing was made by a native speaker.

--------------------


•       The conclusion does not refer to the relevance of study findings to promote and recommend actions to access antivenoms soon after a snakebite occurs and to bring traditional medicine and Western medical treatment together. As stated in lines 484-487.

RESPONSE: Conclusion was changed accordingly.
